# Loop-mediated isothermal amplification (LAMP) assay for specific and rapid detection of *Dickeya fangzhongdai* targeting a unique genomic region

**DOI:** 10.1038/s41598-022-22023-4

**Published:** 2022-11-10

**Authors:** Anuhea DeLude, Riley Wells, Sherine Boomla, Shu-Cheng Chuang, Frank Urena, Aaron Shipman, Noelle Rubas, Donna Lee Kuehu, Buster Bickerton, Taylor Peterson, Shefali Dobhal, Dario Arizala, Diksha Klair, Francisco Ochoa-Corona, Md Emran Ali, Jenee Odani, Jon-Paul Bingham, Daniel M. Jenkins, Jacqueline Fletcher, James P. Stack, Anne M. Alvarez, Mohammad Arif

**Affiliations:** 1 Department of Plant and Environmental Protection Sciences, University of Hawaii at Manoa, Honolulu, HI USA; 2 Department of Molecular Biosciences and Bioengineering, University of Hawaii at Manoa, Honolulu, HI USA; 3 Department of Human Nutrition, Food and Animal Sciences, University of Hawaii at Manoa, Honolulu, HI USA; 4 Institute for Biosecurity & Microbial Forensics, Oklahoma State University, Stillwater, OK USA; 5 Department of Plant Pathology, University of Georgia, Tifton, GA USA; 6 Department of Plant Pathology, Kansas State University, Manhattan, KS USA; 7 Department of Cell and Molecular Biology, University of Hawaii at Manoa, Honolulu, HI USA

**Keywords:** Microbiology, Plant sciences

## Abstract

*Dickeya fangzhongdai*, a bacterial pathogen of taro (*Colocasia esculenta*), onion (*Allium* sp.), and several species in the orchid family (*Orchidaceae*) causes soft rot and bleeding canker diseases. No field-deployable diagnostic tool is available for specific detection of this pathogen in different plant tissues. Therefore, we developed a field-deployable loop-mediated isothermal amplification (LAMP) assay using a unique genomic region, present exclusively in *D. fangzhongdai*. Multiple genomes of *D. fangzhongdai*, and other species of *Dickeya*, *Pectobacterium* and unrelated genera were used for comparative genomic analyses to identify an exclusive and conserved target sequence from the major facilitator superfamily (MFS) transporter gene region. This gene region had broad detection capability for *D. fangzhongdai* and thus was used to design primers for endpoint PCR and LAMP assays. In-silico validation showed high specificity with *D. fangzhongdai* genome sequences available in the NCBI GenBank genome database as well as the in-house sequenced genome. The specificity of the LAMP assay was determined with 96 strains that included all *Dickeya* species and *Pectobacterium* species as well as other closely related genera and 5 hosts; no false positives or false negatives were detected. The detection limit of the assay was determined by performing four sensitivity assays with tenfold serially diluted purified genomic DNA of *D. fangzhongdai* with and without the presence of crude host extract (taro, orchid, and onion). The detection limit for all sensitivity assays was 100 fg (18–20 genome copies) with no negative interference by host crude extracts. The assays were performed by five independent operators (blind test) and on three instruments (Rotor-Gene, thermocycler and dry bath); the assay results were concordant. The assay consistently detected the target pathogen from artificially inoculated and naturally infected host samples. The developed assay is highly specific for *D. fangzhongdai* and has applications in routine diagnostics, phytosanitary and seed certification programs, and epidemiological studies.

## Introduction

An emerging bacterial pathogen threatens taro (*Colocasia esculenta*), onion (*Allium* sp*.*), and various species in the orchid family (*Orchidaceae*). Taro is among the world's essential starch crops needed for food security, especially in Asia, Africa, and the Pacific Islands, with an estimated production value of $4.1 billion dollars in 2016^1–3^. In 2017, gross global onion production was valued at $44.7 billion^2^. Orchids are a growing commodity in global horticultural trade contributing to the economic development of tropical Asia. These crops represent significant value to the agricultural economies of the world, necessitating improved crop protection measures during production and marketing. Recently, *Dickeya fangzhongdai* was associated with soft rot diseases among these economically important plant species and new occurrences of this pathogen have been reported in several regions^4–7^.

*Dickeya fangzhongdai* is classified within *Pectobacteriaceae*, a genetically diverse, Gram-negative, facultatively anaerobic bacterial species capable of causing soft rot disease in a range of monocot hosts and also reported to cause bleeding canker disease of pear (*Pyrus pyrifolia*), a dicot host^8–12^. *Dickeya* species may persist in soil, surface water, and groundwater, or as epiphytes and saprophytes^13–16^. Infection by *Dickeya* species begins with attraction, adhesion, and invasion of the host through wounds and natural openings such as stomata. Plant cell wall-degrading enzymes (PCWDE) initiate dissolution of hemicelluloses, a major component of the middle lamella, resulting in further dissolution of cell walls^17^. Symptoms are not always obvious at initial stages of the disease, and its progress is dependent on the rate of bacterial multiplication as well as environmental conditions such as temperature, humidity, and the availability of oxygen^18^.

Ideally, regulated pathogens would be detected at borders to prevent disease transmission across cropping areas during trading. Biosecurity protocols for crop management require accurate and robust diagnostic assays to prevent and/or mitigate the spread of economically damaging pathogens^19^. Diagnostic assays must consider the biological interactions between pathogens, their hosts, and the environment for robust analyte detection and confidence in assay results. Therefore, natural conditions for infection and sample types, i.e., DNA isolation matrix, niche habitat, and pathogen biology, are crucial for the development of reliable diagnostic tools^20^. Reliable target sensitivity at critically low detection limits is essential for the field application of diagnostic strategies, especially with regards to inherent fluctuation patterns in pathogen abundance over the course of the disease cycle. A robust field implementable diagnostic tool should adequately address inhibitory factors and non-target components of natural isolation matrices to maintain high target sensitivity while also guaranteeing target specificity^21^.

While endpoint and quantitative PCR are among the few diagnostic techniques currently available for detecting *Dickeya* spp. and *D. fangzhongdai* in agricultural food crops^22^, these methods are impractical for field application. Loop mediated isothermal amplification (LAMP) technology offers a practical solution for field-deployable diagnostics that requires less time and space for point-of-need testing, additional capabilities to laboratories that lack specialized diagnostic equipment^23^. This diagnostic technology can be implemented globally to supplement biosecurity and disease management programs, especially in regions agriculturally dependent on onion, taro, orchid, and other susceptible crops. LAMP assays are highly sensitive and efficient for detection of nucleic acid sequences and can produce results in thirty minutes or less^24,25^. As an isothermal reaction, LAMP can facilitate target amplification from crude DNA extracts using only a heating block or water bath^23,26^. A suite of primers guides the polymerase to create several stem-loop structures of varying lengths, resulting in a high concentration of the target sequence. Several methods are used to visualize the results of a LAMP reaction, ranging from colorimetric indicators to gel electrophoresis, which allows adaptation of this protocol to diverse diagnostician’s needs^23,25,27^. Multiplex reactions are possible with LAMP due to its compatibility with lateral flow devices^28^ and fluorescent probes^29^. Rapid and accurate diagnosis is necessary for efficient disease management, and LAMP results can be determined in less than half an hour^25,27^. The simplicity, specificity, sensitivity, and speed of LAMP make this method an ideal point-of-need diagnostic tool.

The objective of this study was to develop a highly specific and sensitive LAMP assay to readily test for the target pathogen in field environments. Four target primers and two loop primers were developed to detect a region of the conserved major facilitator superfamily (MFS) transporter gene region unique to *D. fangzhongdai* and thus detect this pathogen in infected crops. The primers were verified as specific to *D. fangzhongdai* when tested against closely related species. The developed LAMP assay has potential applications in point-of-need plant disease diagnostics, farm management, disease surveys, and plant biosecurity.

## Results

### Target selection and primer design

**Figure 1:**
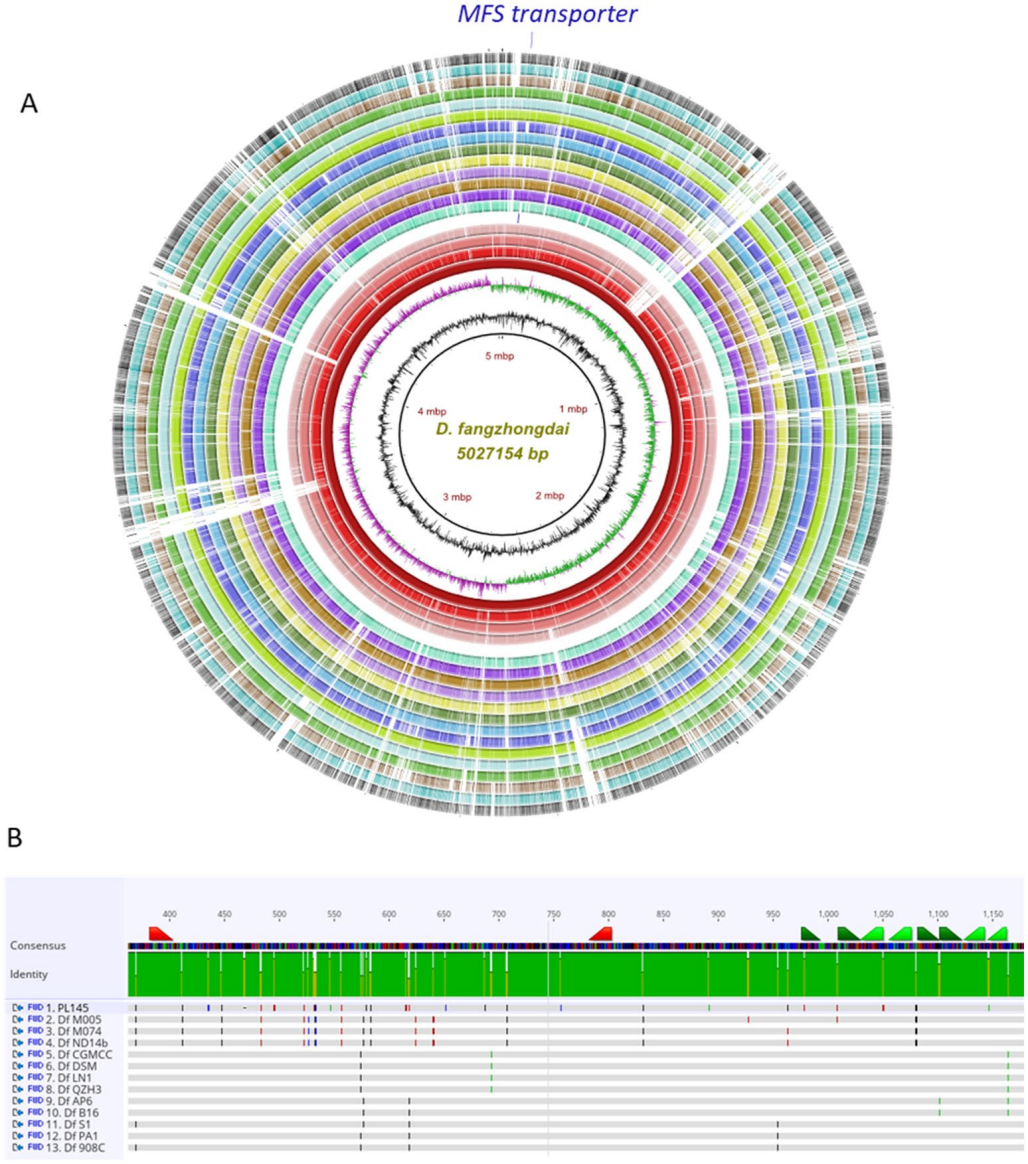
(**A**) Circular graphic pointing out the location of the target sequence MFS transporter exclusively present in *Dickeya fangzhongdai*. The ring image illustrates the multiple genome alignment of four *D. fangzhongdai* strains followed by the other eleven species that currently form *Dickeya*. The last three outermost rings depict the genomes of three different species within *Pectobacterium*. The three innermost rings depict the genome coordinates in terms of mega base pairs (Mbp), the GC content (black line in zigzag shape), and the GC skew (zigzag purple +/green −) of the reference genome *D. fangzhongdai* QZH3. The remaining color-coded rings portray, beginning with the innermost ring, the BLASTn pairwise comparison of *D. fangzhongdai* QZH3 (NZ_CP031507), *D. fangzhongdai* ND14b (NZ_CP009460), *D. fangzhongdai* PA1 (NZ_CP020872), *D. fangzhongdai* PL145 (unpublished), position of the target gene encoding for a MFS transporter (DYD83_00310, highlighted and labeled in violet color), *D. aquatica* 174/2^T^ (NZ_LT615367), *D. chrysanthemi* Ech1591 (NC_012912), *D. dadantii* NCPPB 2976^T^ (NZ_CM001978), *D. dianthicola* ME23 (NZ_CP031560), *D. lacustris* S29^T^ (QNUT00000000), *D. oryzae* ZYY5^T^ (SULL00000000), *D. parazeae* Ech586 (NC_013592), *D. poaceiphila* NCPPB 569^T^ (NZ_CP042220), *D. solani* IPO 2222 (NZ_CP015137), *D. undicola* 2B12 (JSYG00000000), *D. zeae* PL65 (NZ_CP040817), *P. parmentieri* RNS 08-42-1A^T^ (NZ_CP015749), *P. brasiliense* 1692 (NZ_CP047495) and *P. carotovorum* XP-13 (NZ_CP063242). The image was created using the BLAST Ring Image Generator (BRIG) v 0.95^40^. (**B**) Alignment of MFS Transporter regions of 13 genomes of *D. fangzhongdai* and endpoint PCR primers in Geneious Prime. Red represents primers used for endpoint PCR while green represents primers used for LAMP. Gray represents identical regions while all other colors indicate a single nucleotide polymorphism compared to the consensus identity.

**Figure 2:**
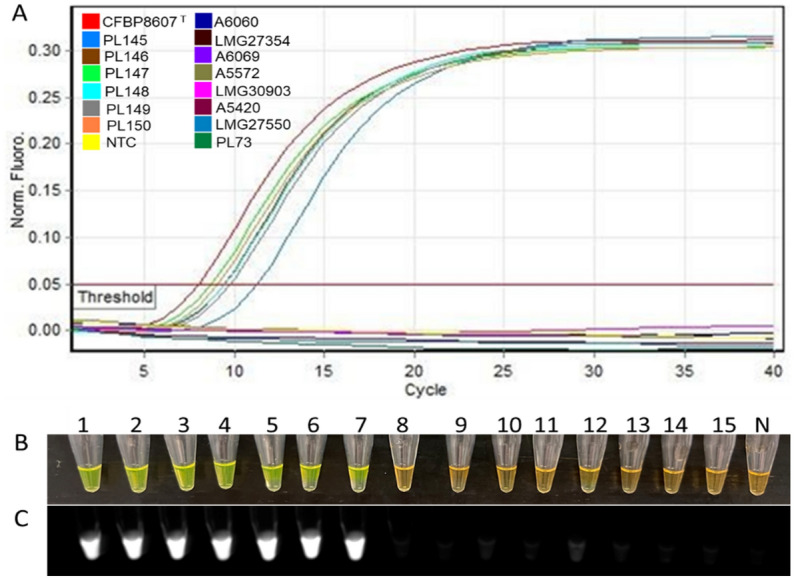
Specificity validation of the loop mediated isothermal amplification (LAMP) primer set designed to amplify a unique MFS transporter gene region of the *Dickeya fangzhongdai*. (**A**) Sigmoidal amplification curves were produced by seven *D. fangzhongdai* strains that formed the inclusivity panel: CFBP8607^T^ (positive control) and PL145, PL146, PL147, PL148, PL149, and PL150. No amplification was observed with gDNA from the strains of the exclusivity panel and non-template control (NTC; water). (**A**,**B**) Tube 1: CFBP8607^T^; tubes 2–7: *D. fangzhongdai* strains, PL145, PL146, PL147, PL148, PL149, and PL150; tubes 8–15: representative strains of the exclusivity panel: LMG27354 (*D. aquatica*), A6060 (*D. dadantii*), A6069 (*D. zeae*), A5572 (*D. dianthicola*), LMG30903 (*D. undicola*), A5420 (*D. paradisiaca*), PL88 (*D. solani*), PL73 (*Pectobacterium carotovorum*); and tube N: NTC. All reactions of gDNA template extracted from bacterial strains of the inclusivity panel produced positive results, while all reactions of the exclusivity panel failed to produce amplification. (**B**) Colorimetric detection of LAMP reaction products under ambient light after the addition of SYBR Green I dye; positive results appear green. (**C**) Detection of the fluorescent emission from the tubes with positive amplification under UV light.

Several potential genomic regions were evaluated for target selection. Regions that were not present in all 13 *D. fangzhongdai* genomes, including a strain from Hawaii that was not previously reported, were not considered for further LAMP assay development. During endpoint PCR validation tests, 4 regions appeared conserved in *D. fangzhongdai* alone based on preliminary specificity tests using CFBP8607^T^ (*D. fangzhongdai* type strain), PL145 (*D. fangzhongdai*), PL47 (*D. zeae*), A5307 (*D. oryzae*), LMG30899 (*Dickeya* sp*.*), PL25 (*D. dianthicola*), and PL84 (*D. solani*) (data not shown). Later, each region was evaluated in silico using the NCBI genome database. The major facilitator superfamily (MFS) transporter gene region was selected as a highly unique region in *D. fangzhongdai* and used for development of a specific and robust assay (Fig. 1). This region was not identified in other *Dickeya* species nor in the closely related genus, *Pectobacterium*. When tested using an endpoint PCR at an annealing temperature of 57 °C, MFS transporter region primers did not have off-target amplification. In-silico analysis indicated that the 1409-nucleotide long MFS transporter region was not identical in other genomes present in the NCBI GenBank genome database. However, the MFS transporter region was found in all genomes of *D. fangzhongdai* publicly available as well as one unpublished genome of a *D. fangzhongdai* strain isolated in Hawaii (Table 1). Results from BLAST database revealed a high identity among all strains of *D. fangzhongdai* for this region, where query coverage was above 99% and pairwise identity was above 96.5% compared to the genome (NZ_CP025003.1) of the type strain CFBP8607^T^ (Table 1). Endpoint primers and six LAMP primers, designed using the MFS transporter gene, were specific to all *D. fangzhongdai* genomes available, showing no significant identity with other genomes in the NCBI GenBank database. Endpoint primers have a 100% sequence match to all strains listed in Table 1, and LAMP primers have a 100% sequence match with all genomes obtained from the NCBI GenBank, although 2 SNPs among 6 primers were observed for the strain isolated in Hawaii (Table 2; Fig. 1B). The SNPs did not impact the specificity of the assay (Table 3; Fig. 2).

**Table 1:** Custom BLAST database results for *Dickeya fangzhongdai* MFS transporter gene region in all available genomes (draft and complete) retrieved from NCBI GenBank as well as one unpublished genome isolated in Hawaii (strain PL145).

Strain name	Host	Location	GenBank accession no.	E-value	Pairwise identity (%)	Identical sites (%)	Query cover (%)
QZH3	Pear	China	NZ_CP031507	0	100	100	100
CGMCC 1.15464	N/A	China	NZ_BMJF01000013	0	100	100	100
LN1	Pear	China	NZ_CP031505	0	100	100	100
PA1	Orchid	China	NZ_CP020872	0	99.3	99.3	100
AP6	Onion	USA	VSRM01000004	0	99.1	99.1	100
908C	Orchid	Canada	NZ_JADCNJ010000021	0	99.2	99.1	100
S1	Orchid	Slovenia	JXBO02000014	0	99.1	99.2	99.36
B16	Orchid	Slovenia	NZ_JXBN02000007	0	97.9	99.1	99.36
M074	Waterfall	Malaysia	JRWY01000043	0	97.9	97.9	100
ND14b	Waterfall	Malaysia	NZ_CP009460	0	97.9	97.9	100
M005	Waterfall	Malaysia	JSXD01000006	0	97.7	97.7	100
PL145	Taro	USA	N/A	0	96.5	96.5	99

The MFS transporter gene sequence of *D. fangzhongdai* type strain CFBP8607^T^ was used for comparison.

**Table 2:** Details of primers used for endpoint PCR and loop-mediated isothermal amplification (LAMP) assays for detection of *Dickeya fangzhongdai*.

Primer name	Sequence (5′–3′)	Length (nt)	GC%	Assay type
AD-F	CGCAATACGCCTAAAGATCAA	21	42.9	Endpoint PCR
AD-R	GAAAGGTCGGCACATTCAGT	20	50.0	
Df-F3	CGCCGTTTCGGCTTCAA	17	58.8	LAMP
Df-B3	CCGGAACATCCGCGAAC	17	64.7	
Df-FIP	GAATCTGCGGCGTGGTGGGG-AACGGCATACTGTGCGTATT	40	56.1	
Df-BIP	TATTGCTGCTGTTCTGGGG-AGCGAACTGATGCCGGTAA	38	51.3	
Df-LF	GCGGCACAAAGCAGCAACGA	20	60.0	
Df-LB	GGACTCAGCCGTTCGATGCA	20	60.0	

The MFS transporter gene region of *D. fangzhongdai* was used to design these primers.

**Table 3:** Details of bacterial strains, healthy plant hosts and environmental sources, included in inclusive and exclusive panels, used for the validation of *Dickeya fangzhongdai* specific loop-mediated isothermal amplification (LAMP) assay.

Species name	Strain ID	Geographic location	Host/source	LAMP result
**Inclusivity panel**				
*Dickeya fangzhongdai*	CFBP8607^T^	China	*Pyrus pyrifolia* (Asian pear)	Positive
*D. fangzhongdai*	PL145	Hawaii, USA	*Colocasia esculenta* (Taro)	Positive
*D. fangzhongdai*	PL146	Hawaii, USA	*C. esculenta* (Taro)	Positive
*D. fangzhongdai*	PL147	Hawaii, USA	*C. esculenta* (Taro)	Positive
*D. fangzhongdai*	PL148	Hawaii, USA	*C. esculenta* (Taro)	Positive
*D. fangzhongdai*	PL149	Hawaii, USA	*C. esculenta* (Taro)	Positive
*D. fangzhongdai*	PL150	Hawaii, USA	*C. esculenta* (Taro)	Positive
**Exclusivity panel**				
*D. aquatica*	LMG27354	UK	River water	Negative
*D. chrysanthemi*	CFBP2048/A5415	USA	*Chrysanthemum morifolium* (China juhua)	Negative
*D. chrysanthemi*	CFBP1270/A5641	Denmark	*Parthenium argentatum* (Guayule)	Negative
*D. dadantii*	CFBP3698/ A6060	Cuba	*Musa* sp. (Banana)	Negative
*D. dianthicola*	A5572	Netherlands	*Solanum tuberosum* (Potato)	Negative
*D. dianthicola*	PL22	Hawaii, USA	*S. tuberosum* (Potato)	Negative
*D. dianthicola*	PL25	Hawaii, USA	*S. tuberosum* (Potato)	Negative
*D. dianthicola*	CFBP2015	France	*S. tuberosum* (Potato)	Negative
*D. lacustris*	LMG30899	France	Water	Negative
*D. oryzae*	A5304	Hawaii, USA	*Ananas comosus* (Pineapple)	Negative
*D. oryzae*	A5307	Hawaii, USA	*A. comosus* (Pineapple)	Negative
*D. oryzae*	A5310	Hawaii, USA	*A. comosus* (Pineapple)	Negative
*D. zeae*	CFBP 1278	Malaysia	*A. comosus* (Pineapple)	Negative
*D. solani*	LMG27549	Ireland	*S. tuberosum* (Potato)	Negative
*D. solani*	LMG27550	Finland	*S. tuberosum* (Potato)	Negative
*D. solani*	LMG27554	Poland	*S. tuberosum* (Potato)	Negative
*D. solani*	LMG25865	Belgium	*S. tuberosum* (Potato)	Negative
*D. solani*	LMG25993	Netherlands	*S. tuberosum* (Potato)	Negative
*D. undicola*	LMG30903	Malaysia	Fresh water	Negative
*D. zeae*	A5629	Hawaii, USA	Irrigation water	Negative
*D. zeae*	A6069	USA	*Zea mays* (Maize)	Negative
*D. zeae*	PL47	Hawaii, USA	*Brassica oleracea* (Kale)	Negative
*D. zeae*	PL65	Hawaii, USA	*C. esculenta* (Taro)	Negative
*D. zeae*	CFBP2052/A5422	USA	*Z. mays* (Maize)	Negative
*D. zeae*	CFBP6466/A5423	Martinique	*A. comosus* (Pineapple)	Negative
*Dickeya* sp.	A5279	Hawaii, USA	Irrigation water	Negative
*D. zeae*	A5410	Hawaii, USA	*A. comosus *(Pineapple)	Negative
*Pectobacterium actinidiae*	LMG26003	South Korea	*Actinidia chinensis* (Kiwi)	Negative
*P. actinidiae*	LMG26004	South Korea	*A. chinensis* (Kiwi)	Negative
*P. aquaticum*	CFBP8637	France	Environment/fresh water	Negative
*P. aroidearum*	LMG2414	Israel	*Persea americana* (Avocado)	Negative
*P. aroidearum*	LMG2417	South Africa	*Zantedeschia aethiopica* (Calla Lily)	Negative
*P. atrosepticum*	A1654	−	−	Negative
*P. atrosepticum*	LMG2386	UK	*S. tuberosum* (Potato)	Negative
*P. atrosepticum*	LMG2374	UK	*Apium graveolens* var. *dulce* (Celery)	Negative
*P. atrosepticum*	LMG2375	UK	*S. tuberosum* (Potato)	Negative
*P. betavasculorum*	LMG2461	USA	*Beta vulgaris* (Beet)	Negative
*P. betavasculorum*	LMG2466	USA	*B. vulgaris* (Beet)	Negative
*P. betavasculorum*	wis_A3000	−	−	Negative
*P. brasiliense*	PL48	Hawaii, USA	*B. oleracea* (Kale)	Negative
*P. brasiliense*	PL63	Hawaii, USA	*B. oleracea* (Kale)	Negative
*P. brasiliense*	PL64	Hawaii, USA	*B. oleracea* (Kale)	Negative
*P. brasiliense*	PL68	Hawaii, USA	*S. tuberosum* (Potato)	Negative
*P. brasiliense*	PL243	Hawaii, USA	*Brasica rapa* var *chinensis* (Pakchoi)	Negative
*P. brasiliense*	LMG21371	Brazil	*S. tuberosum* (Potato)	Negative
*P. cacticida*	LMG17936	Arizona, USA	*Carnegiea gigantea* (Saguaro)	Negative
*P. carotovorum*	PL73	Hawaii, USA	*S. tuberosum* (Potato)	Negative
*P. cypripedii*	LMG1268	USA	*Cypripedium* sp. (Orchid)	Negative
*P. fontis*	LMG30744	Malaysia	Fresh water	Negative
*P. parvum*	CFBP 8631	Finland	*S. tuberosum* (Potato)	Negative
*P. parmentieri*	LMG29774	France	*S. tuberosum* (Potato)	Negative
*P. polaris*	ICMP 9180	Netherlands	*S. tuberosum* (Potato)	Negative
*P. polonicum*	LMG31077	Poland	Groundwater from potato fields	Negative
*P. punjabense*	LMG30622	Pakistan	*S. tuberosum* (Potato)	Negative
*P. versatiles*	PL62	Hawaii, USA	*S. tuberosum* (Potato)	Negative
*P. versatiles*	ICMP 9168	Netherlands	*S. tuberosum* (Potato)	Negative
*P. wasabiae*	wis_A1438/CFBP3304	Japan	*Eutrema wasabi* (Wasabi)	Negative
*P. zantedeschiae*	CFBP1357	France	*Zantedeschia* sp. (Aroid)	Negative
*Pectobacterium* sp.	PL34	Hawaii, USA	*Hoodia* sp.	Negative
*Pectobacterium* sp.	PL151	Hawaii, USA	*C. esculenta* (Taro)	Negative
*Pectobacterium* sp.	PL152	Hawaii, USA	*C. esculenta* (Taro)	Negative
*Pectobacterium* sp.	PL153	Hawaii, USA	*C. esculenta* (Taro)	Negative
*Pectobacterium* sp.	PL154	Hawaii, USA	*C. esculenta* (Taro)	Negative
*Pectobacterium* sp.	PL155	Hawaii, USA	*C. esculenta* (Taro)	Negative
*Pectobacterium* sp.	PL156	Hawaii, USA	*C. esculenta* (Taro)	Negative
*Pectobacterium* sp.	PL157	Hawaii, USA	*C. esculenta* (Taro)	Negative
*Pectobacterium* sp.	PL158	Hawaii, USA	*C. esculenta* (Taro)	Negative
*Pectobacterium* sp.	PL160	Hawaii, USA	*C. esculenta* (Taro)	Negative
*Pseudomonas* sp.	PL159	Hawaii, USA	*C. esculenta* (Taro)	Negative
*Clavibacter sepedonicus*	A2041	Denmark	*S. tuberosum* (Potato)	Negative
*C. nebraskensis*	A6205	Iowa, USA	*Z. mays* (Maize)	Negative
*Pantoea agglomerans*	A6222	Wisconsin, USA	*Z. mays* (Maize)	Negative
*Pantoea* sp.	A1867	Hawaii, USA	*Carica papaya* (Papaya)	Negative
*Pantoea* sp.	A6219	USA	*Z. mays* (Maize)	Negative
*C. nebraskensis*	DP164/A6212	USA	*Z. mays* (Maize)	Negative
*Enterobacter cloacae*	A5149	Hawaii, USA	*Zingiber officinale* (Ginger)	Negative
*E. homaechei*	PL169	Hawaii, USA	*B. oleracea* (Kale)	Negative
*Enterobacter* sp.	PL168	Hawaii, USA	*B. oleracea* (Kale)	Negative
*Erwinia amylovora*	A1084	−	*Pyrus* sp. (Pear)	Negative
*Klebsiella* sp.	A223	−	*Vanda* sp. (Orchid)	Negative
*Klebsiella* sp.	A6223	Iowa, USA	*Z. mays* (Maize)	Negative
*Microbacterium* sp.	A6214	Iowa, USA	*Z. mays* (Maize)	Negative
*Musicola paradisiaca*	CFBP4178/A5420*	Colombia	*M. paradisiaca* (Banana)	Negative
*Ralstonia solanacearum*	19170	Guam, USA	*Casuarina equisetifolia* (Ironwood)	Negative
*Rathayibacter rathayi*	ATCC 13659/A1152	UK	Grass	Negative
*R. tritici*	LMG3726/A6287	Egypt	*Triticum aestivum*	Negative
*Xanthomonas arboricola*	A1964	Florida, USA	*Anthurium* sp. (Aroid)	Negative
*X. phaseoli* pv. *dieffenbachiae*	D119	Hawaii, USA	*Anthurium* sp. (Aroid)	Negative
*X. phaseoli* pv. *dieffenbachiae*	D182	Hawaii, USA	*Anthurium* sp. (Aroid)	Negative
**Exclusivity panel (plant/soil samples)**				
*Allium cepa*	Healthy Onion	Hawaii, USA	−	Negative
*Colocasia esculenta*	Healthy Taro	Hawaii, USA	−	Negative
*Phalaenopsis* sp.	Healthy Orchid	Hawaii, USA	−	Negative
*Solanum tuberosum*	Healthy Potato	Hawaii, USA	−	Negative
–	Non-infested soil	Hawaii, USA	−	Negative

Negative (−) signs indicate data not available. ^T^indicates type strain. *Previously known as *Dickeya paradisiaca*; Hugouvieux-Cotte-Pattat et al.^41^ proposed the species belongs to a newly proposed genus, *Musicola.*

### Specificity assays

The specificity of the LAMP primers was validated using selected inclusivity and exclusivity panels. The inclusivity panel consisted of purified genomic DNA (gDNA) extracted from seven *D. fangzhongdai* strains, while the exclusivity panel consisted of purified gDNA extracted from 89 non-target bacterial strains of different species (Table 3). The exclusivity panel also included DNA of four plant hosts and one soil sample (Table 3). The assay successfully detected all *D. fangzhongdai* strains in the inclusivity panel, CFBP8607^T^ and PL145-PL150, while no false positives were obtained from non-target bacterial species, host plants, or soil (Table 3). Based on melting curve results, the single peak result indicated all seven *D. fangzhongdai* strains presenting positive calls as well (Fig. S1). Results were visualized by real time analysis of sigmoidal amplification curves (Rotor-Gene Q), and colorimetrically after adding SYBR Green I dye^30^. During amplification, the cycle threshold of positive calls ranged from 7 to 10. After the addition of SYBR Green I dye, tubes with positive reactions turned green under ambient daylight and emitted fluorescence upon excitation with UV light. The results of all seven *D. fangzhongdai* strains were similar when resolved under the described visualization methods, whereas none of the 94 DNA samples in the exclusivity panel were amplified, evident by real time analysis, the orange appearance under ambient light, and the lack of fluorescence after the addition of SYBR Green dye (Table 3, Fig. 2).

**Figure 3:**
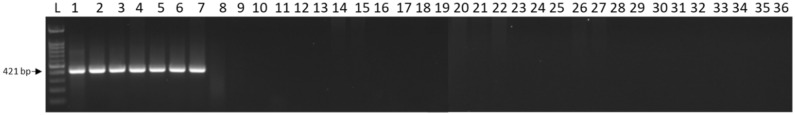
The specificity validation of the endpoint PCR primer pair designed to specifically detect *Dickeya fangzhongdai*. A total of 36 reactions including seven *D. fangzhongdai:* CFBP8607^T^ (positive control, lane 1) and PL145-PL150 (lanes 2–7) from the inclusivity panel used for the validation of the LAMP primer set. The remaining lanes consisted of reactions of gDNA templates extracted from 28 representative strains forming the PCR exclusivity panel (lanes 8–35): A6069, A5420, A6060, PL84, A5572, LMG27354, PL73, CFBP8637, LMG2414, 32F, CFBP8631, LMG17936, LMG30899, LMG30903, CFBP1357, LMG2466, LMG1268, LMG21371, LMG30744, LMG26003, 19170, A2041, A6205, A6219, A1084, A5726, A223, and DP164, and non-template control (NTC, lane 36).

The specificity of the endpoint PCR primer pair that was designed to amplify the MFS transporter gene was also validated using the full LAMP inclusivity panel, which was composed of seven *D. fangzhongdai* strains and with a subset of the exclusivity panel used to validate the LAMP assay (Fig. 3). The reduced exclusivity panel consisted of 28 bacterial strains of phylogenetically close genera, and of niche- and host sharing-species; the panel included non-target *Dickeya* species, *Pectobacterium*, *Ralstonia*, *Xanthomonas*, and other Gram-positive bacteria, such as *Clavibacter* and *Rathayibacter* species (Fig. 3). All PCR reactions were completed by using the same gDNA sources that were used for the LAMP exclusivity assay. Reactions with *D. fangzhongdai* gDNA templates yielded the expected amplification product, 421-bp in length, while reactions completed using template DNA from other selected species did not produce any amplification products apparent during resolution of PCR products by agarose gel electrophoresis (Fig. 3).

### Limit of detection and spiked assays

**Figure 4:**
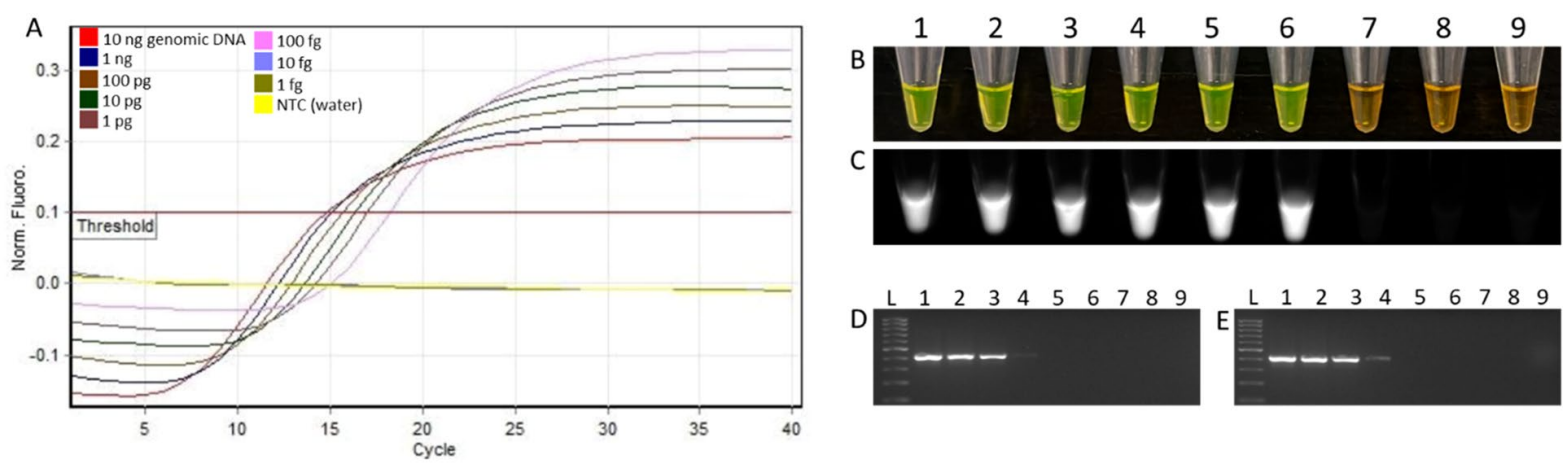
Detection limit determination of loop mediated isothermal amplification (LAMP) assay designed for specific detection of *Dickeya fangzhongdai.* Purified genomic DNA (CFBP8607^T^), serially diluted tenfold (from 10 ng to 1 fg) was used per reaction (tubes 1–8), with tube 9 being non-template control (NTC) water. (**A**) Sigmoidal curves; (**B**) results after adding SYBR Green I dye to the amplified reaction—a color change from orange to bright green indicates a positive amplification; (**C**) tubes observed under UV light—fluorescence is indicative of positive amplification. Positive amplification with the LAMP assay was observed for concentrations as little as 100 fg (tube 6). (**D**) Endpoint sensitivity assay with genomic DNA, serially diluted tenfold (from 10 ng to 1 fg); (**E**) spiked assay (1 µl of healthy taro corm genomic DNA was added in tenfold serially diluted genomic DNA). In both cases, endpoint PCR was 100-times less sensitive than the LAMP assay.

**Figure 5:**
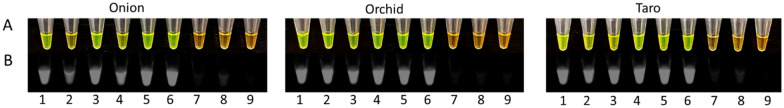
Detection limit determination of the loop mediated isothermal amplification (LAMP) assay designed for specific detection of *Dickeya fangzhongdai* in the presence of host matrices*.* Five µl of crude host lysate was added to purified genomic DNA (CFBP8607), tenfold serially diluted (from 10 ng to 1 fg) (tubes 1 to 8; tube 9 was non-template control). (**A**) Addition of SYBR Green I dye—a bright green color indicates positive amplification; (**B**) tubes observed under UV light—fluorescence indicate positive amplification. Amplification was observed for concentrations as little as 100 fg (tube 6), indistinguishable from the limit of detection obtained without the addition of crude host lysate.

The limit of detection of the *D. fangzhongdai* LAMP assay was determined by performing the assay on a tenfold serially diluted preparation of genomic DNA of type strain CFBP8607. The LAMP assay successfully detected the target DNA at a minimum concentration of 100 fg (Fig. 4A–C). To assess possible reaction inhibition by host matrices, three spiked assays (onion, orchid, and taro) were performed by adding 5 µl crude lysate (this 5 µl amount is recommended by the manufacturer when running an assay with plant samples) derived from healthy host tissues to the tenfold serially diluted genomic DNA of *D. fangzhongdai*^23^. Assays spiked with any of the three crude host lysates maintained the 100 fg detection limit (Fig. 5). The inclusion of non-template control (NTC; water) in each reaction reported no occurrence of false positive results. Using Rotor-Gene Q, SYBR Green and UV light to validate the results, no discrepancies were found. When comparing assay sensitivity with endpoint PCR, LAMP was 100-fold more sensitive (Figs. 4, 5).

### Multi-operator blind tests

**Figure 6:**
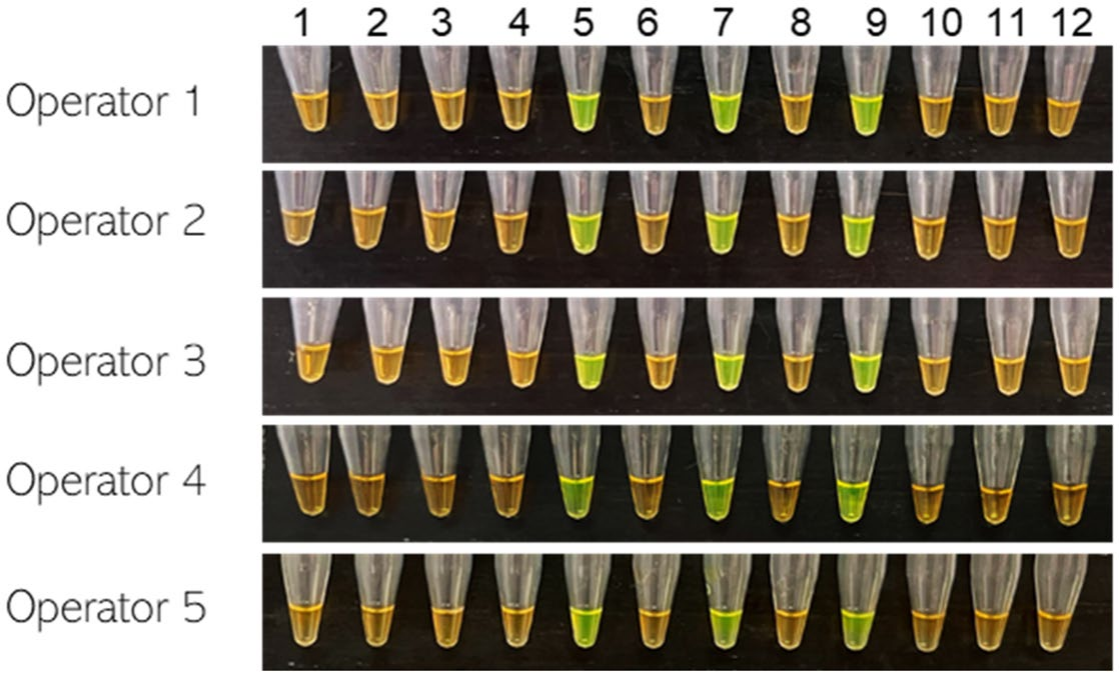
Multi-operator blind tests of the *Dickeya fangzhongdai* specific loop mediated isothermal amplification (LAMP) assay were performed to validate reproducibility and robustness. Twelve representative samples were used: Tubes 1–12: *Dickeya lacustris* LMG30899, *D. dianthicola* PL25, *D. solani* LMG27552, *D. zeae* A5422, *D. fangzhongdai* CFBP8607^T^, *D. aquatica* LMG27354, *D. fangzhongdai* PL146*, Pantoea* sp*.* A1869, *D. fangzhongdai* PL148, non-template control (NTC; water), *D. chrysanthemi* A5641 and *D. paradisiaca* A5579, respectively.

Five different operators performed multi-operator blind tests with twelve blind samples to confirm reproducibility and robustness of the developed assay. All 12 samples, including the NTC, were tested with the LAMP assay to specifically detect three *D. fangzhongdai* strains (Fig. 6). All operator results were 100% in agreement and specifically detected *D. fangzhongdai* (Fig. 6). No false positives or false negatives were detected during the blind test.

### Multi-device detection of *D. fangzhongdai* directly from bacterial colonies

**Figure 7:**
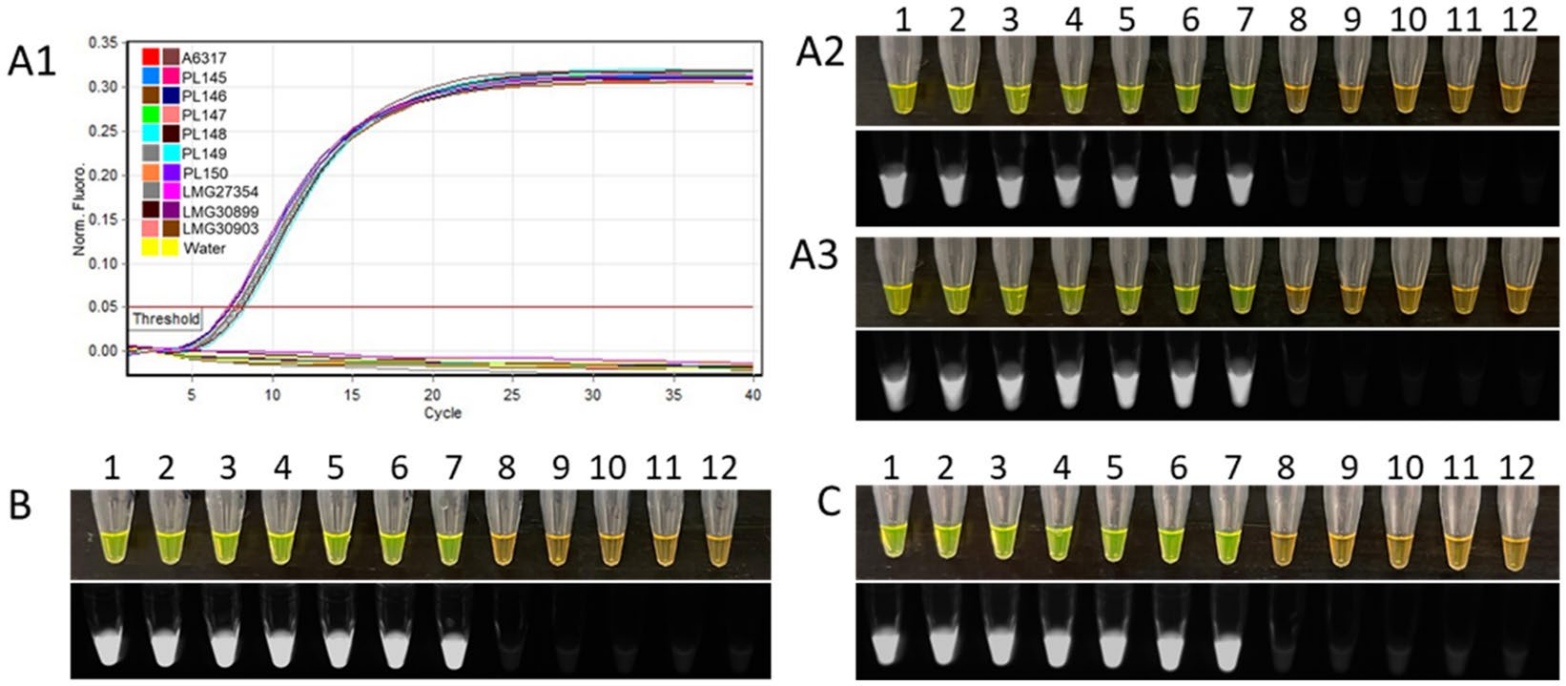
Multi-device detection directly from colonies of *D. fangzhongdai* were validated using three different incubation platforms. Eleven representative samples were tested. Tubes 1–11: *D. fangzhongdai* strains CFBP8607^T^, PL145, PL146, PL147, PL148, PL149, PL150, *D. aquatica* LMG 27354, *D. lacustris* LMG30899, *D. undicola* LMG30903, *D. oryzae* A5410 and non-template negative control (NTC; water), respectively. Three detection methods were used: sigmoidal curves, SYBR Green I dye detected visually, and fluorescence detection under UV light. (**A1**–**A3**) Rotor-Gene amplification reaction performed by two independent groups with positive amplification observed by sigmoidal curve and further visualized by adding SYBR Green Dye—bright green color indicative of positive amplification; (**B**) LAMP amplification using an endpoint thermal cycler and visualized by adding SYBR Green Dye; (**C**) LAMP amplification using a dry bath (hot plate) and visualized by adding SYBR Green Dye.

Multi-device tests were performed using three different platforms (Qiagen Rotor-Gene G, Bio-Rad thermocycler, and dry bath). Twelve samples were tested, seven were *D. fangzhongdai*, four were various *Dickeya* spp. and a non-template control (NTC; water). All seven DNA samples of *D. fangzhongdai* were validated directly from bacterial colonies. Results were 100% in agreement with the sample correlation (Fig. 7). No false positives or false negatives were detected during the validation test.

### Validation using artificially and naturally infected samples

**Figure 8:**
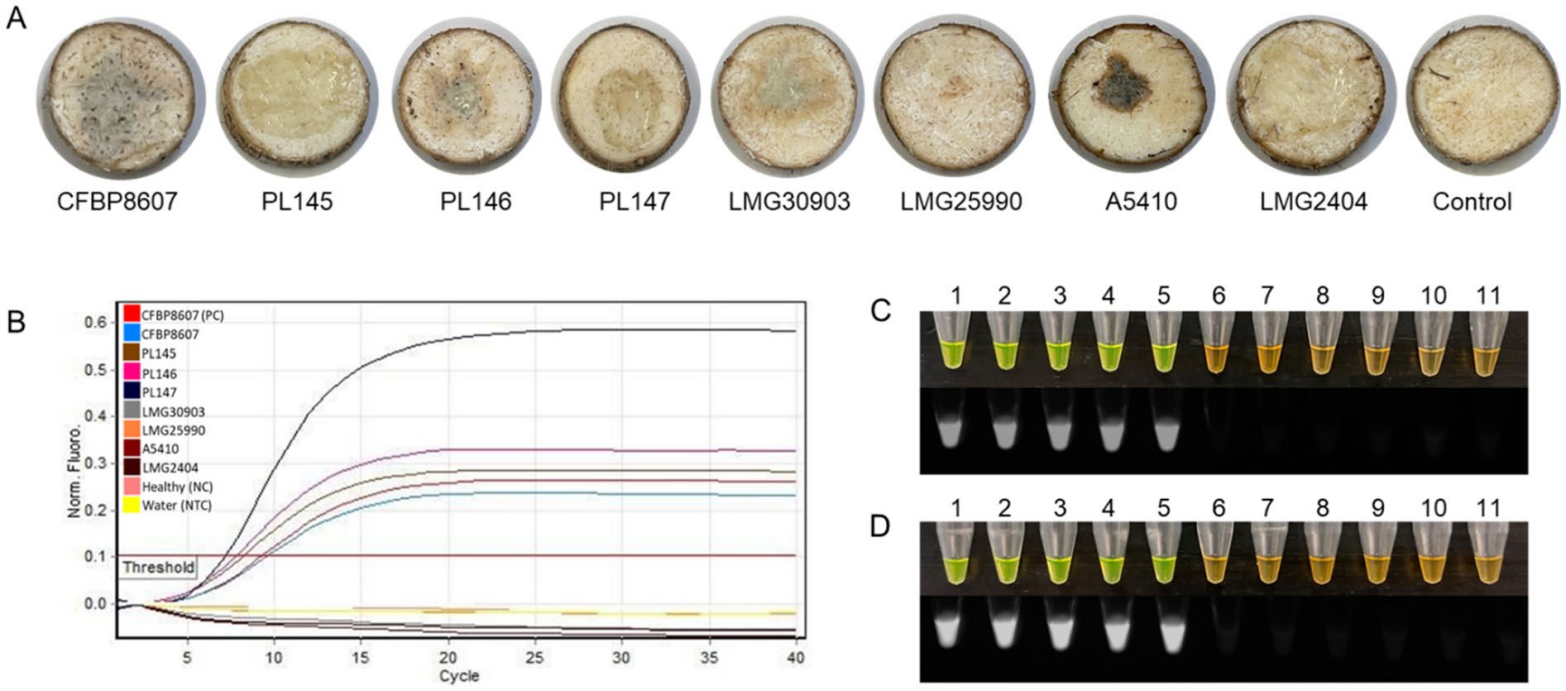
Detection of *D. fangzhongdai* in artificially infected taro samples. (**A**) Taro slices infected with different *Dickeya* spp. and a *Pectobacterium* species; *D. fangzhongdai* CFBP8607^T^, and PL145-PL147, *D. undicola* LMG30903, *D. solani* LMG25990, *D. oryzae* A5410, *P. carotovorum* LMG2404, and control (healthy taro). (**B**) Sigmoid curve diagram—only *D. fangzhongdai* infected taro slices were positive, no curve was observed for other *Dickeya* spp., *P. carotovorum* LMG2404, NC (negative control; healthy taro) and NTC (non-template control; water). (**C**) Rotor-Gene amplification reactions were used for visualization of the LAMP product by adding SYBR Green I dye. Green: positive amplification, orange: no amplification. Visualization of SYBR Green I dye results under UV exposure–fluorescence indicates positive amplification. (**D**) Dry bath amplification reaction was used for visualization of the LAMP product by adding SYBR Green I dye. Visualization of SYBR Green I dye results under UV exposure. 1–2, *D. fangzhongdai* CFBP8607^T^ (purified gDNA and DNA from infected sample); 3–5, *D. fangzhongdai* PL145-PL147; 6–9 *D. undicola* LMG30903, *D. solani* LMG25990, *D. oryzae* A5410, *P. carotovorum* LMG2404; 10–11 healthy taro (NC) and water (NTC).

**Figure 9:**
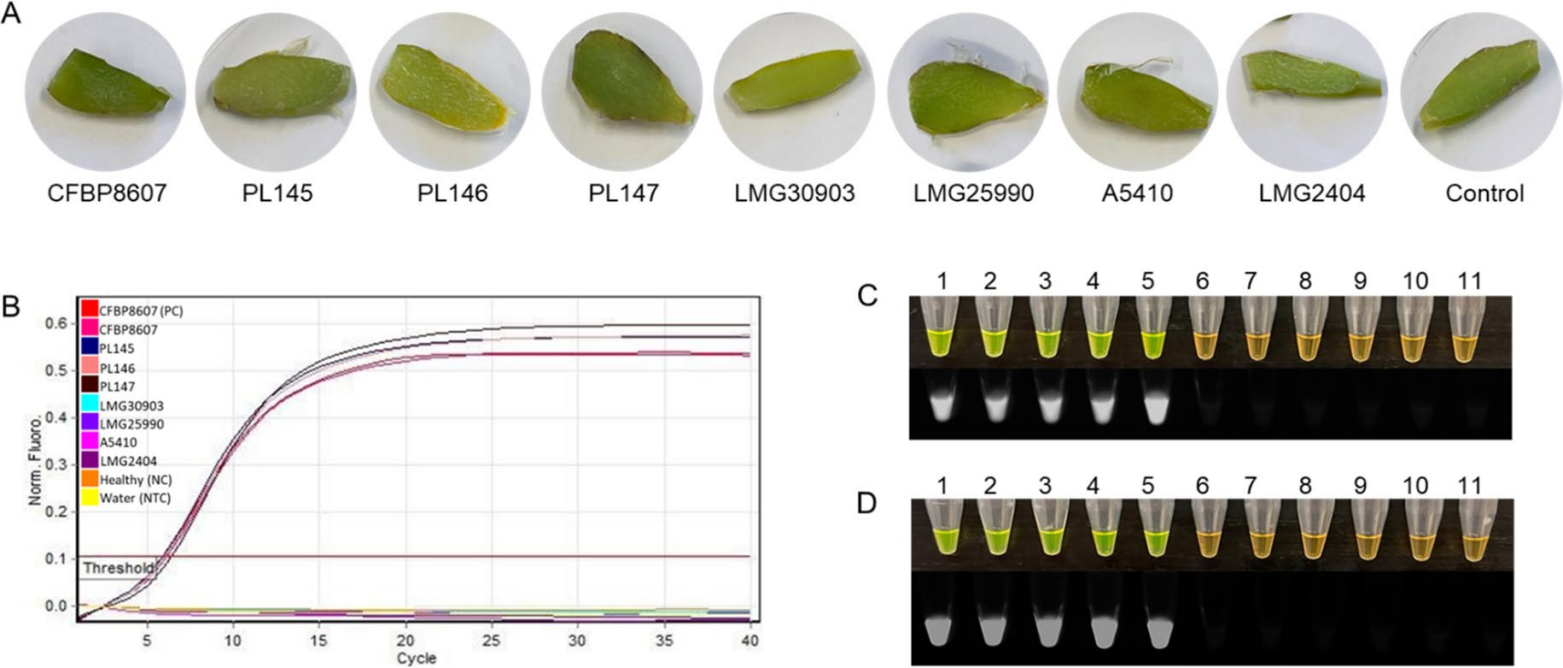
Detection of *Dickeya fangzhongdai* in artificially infected orchid samples. (**A**) Orchid stems infected with different *Dickeya* spp. and a *Pectobacterium* species; *D. fangzhongdai* CFBP8607^T^ and PL145-PL147, *D. undicola* LMG30903, *D. solani* LMG25990, *D. oryzae* A5410, *P. carotovorum* LMG2404, and Control (healthy orchid). (**B**) Sigmoid curve diagram—only *D. fangzhongdai* infected orchid stems were positive, no curve was observed for other *Dickeya* spp., *P. carotovorum* LMG2404, NC (negative control; healthy orchid) and NTC (non-template control; water). (**C**) Rotor-Gene amplification reactions were used for visualization of the LAMP product by adding SYBR Green I dye. Green: positive amplification, orange: no amplification. Visualization of SYBR Green I dye results under UV exposure–fluorescence indicates positive amplification. (**D**) Dry bath amplification reaction was used for visualization of the LAMP product by adding SYBR Green I dye. Visualization of SYBR Green I dye results under UV exposure. 1–2, *D. fangzhongdai* CFBP8607^T^ (purified gDNA and DNA from infected sample); 3–5, *D. fangzhongdai* PL145-PL147; 6–9 *D. undicola* LMG30903, *D. solani* LMG25990, *D. oryzae* A5410, *P. carotovorum* LMG2404; 10–11 healthy orchid (NC) and water (NTC).

**Figure 10:**
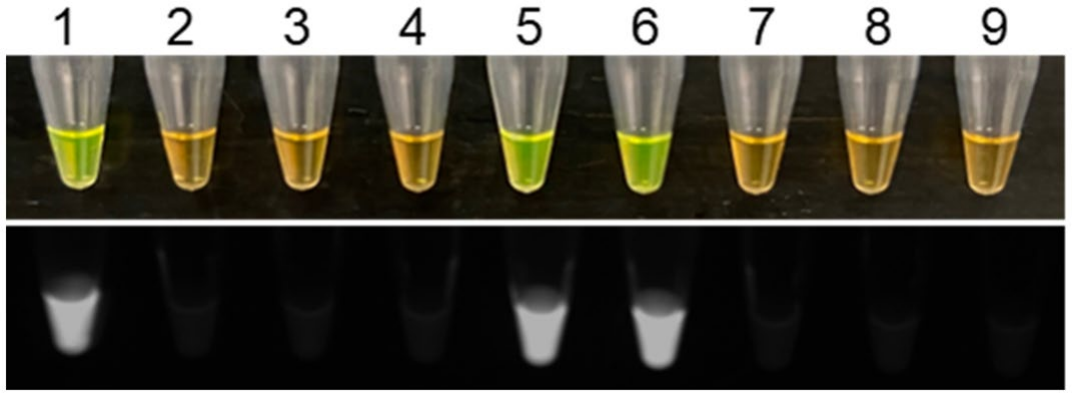
Detection of *Dickeya fangzhongdai* in naturally infected taro samples. Positive amplification was observed with *D. fangzhongdai* CFBP8607^T^ (positive control, tube 1) and two *D. fangzhongdai*-infected taro samples (tubes 5 and 6); no SYBR GREEN color change or fluorescence were observed for non-infected taro samples (tubes 2–4), healthy taro (tube 8) and NTC (non-template control; water; tube 9). No false positives and false negatives were detected.

To further determine its viability as a diagnostic test, the LAMP assay was tested with eight strains of *D. fangzhongdai* by artificially infecting taro corm and orchid stem samples. The LAMP assay accurately determined the presence of *D. fangzhongdai* in four of the samples inoculated with *D. fangzhongdai*, and did not cross-react with any samples inoculated with related *Dickeya* or other microbial species (Figs. 8 and 9). The LAMP assay also accurately determined the presence of *D. fangzhongdai* in two (confirmed by isolating the bacteria; data not shown) of the six taro corm samples from the field, and no amplification with a healthy taro corm sample (Fig. 10). The addition of SYBR Green dye resulted in a color change from orange to green in all samples containing the LAMP product, indicating DNA amplification and a true positive result (Fig. 10). Furthermore, no color change occurred in the non-*D. fangzhongdai* infected/inoculated samples, the negative controls, or healthy samples.

## Discussion

New *Dickeya* species are continually being described due to the broad natural diversity within the genus. To date, descriptions of 12 *Dickeya* species have been published and their genomes are available^15^, including the most recent additions of *D. poaceiphila*^31^, *D. parazeae*^32^, *D. oryzae*^33^ and *D. colocasiae*^16^. A previously published LAMP assay, designed to detect *Dickeya* at the genus level, successfully detected eight known *Dickeya* spp.^34^. The LAMP assay developed in the present study utilized a unique genomic region to provide a rapid, and cost-effective diagnostic assay for specific detection of *D. fangzhongdai* which can be applied easily at point-of-need. The assay was thoroughly validated with all *Dickeya* and *Pectobacterium* species and strains of other bacterial species.

A robust diagnostic assay requires stringent selection of a unique and specific genomic region that is well conserved within the target pathogen genomes^25,30,35^. In silico validation is required to confirm that the target region is present in all strains of the same species and absent in closely related species of *Dickeya* and *Pectobacterium* and other species that occupy the same ecological niche^21^. The unpublished genome of a locally isolated strain of *D. fangzhongdai* (PL145) was used to increase the breadth of in silico analysis, as the publicly available genomes of the species do not currently include Hawaiian strains (Table 1). Four regions were specific to *D. fangzhongdai* based on preliminary in silico genome alignment and analyses, and in vitro endpoint PCR tests (data not shown). High band intensity maintained across different strains of *D. fangzhongdai* and the lack of off-target amplification, even at the relatively low annealing temperature (57 °C), indicated that the MFS transporter was an ideal region for further LAMP assay development. This held true for both sets of primers (endpoint PCR and LAMP assays) designed for this region. LAMP remains an ideal option for diagnostic assay development; it is rapid, comparatively resistant to inhibition, and field deployable^20^. Results can be visualized in several ways, including colorimetric indicators, turbidity, and gel electrophoresis, which enables LAMP to be used effectively in many laboratory settings^25^.

The validation of the designed primers with the members of inclusivity and exclusivity panels is mandatory for confirmation of assay specificity^25^. The positive LAMP results with members of the inclusivity panel shown in Fig. 2; the LAMP assay detected all *D. fangzhongdai* strains—suggest a broad range detection capability of this assay. In the exclusivity panel, clear negative results occurred for the other species of *Dickeya* and *Pectobacterium* (Table 3; Fig. 2). Although two recently described *Dickeya* species, *D. poaceiphila* and *D. parazeae*, were absent from the exclusivity panel, our genomic-informed strategy of selecting the MFS target gene for LAMP development suggests the sequence of the MFS transporter gene is not present in their genomes (Fig. 1A). Hence, the developed LAMP possesses high specificity for distinguishing the species, *D. fangzhongdai*, from phylogenetically closely related species, plant associated niche-sharing species of additional genera, and common habitat matrices. The detection of a target pathogen can be affected adversely by the presence of plant inhibitors, but inhibitors do not affect all assays (developed using different techniques) equally^20,36^. A LAMP assay developed for specific detection of a Gram-positive bacterial pathogen, *Rathayibacter toxicus,* using the OptiGene Master Mix, was less affected by plant inhibitors than an endpoint PCR but more affected than an RPA assay^20^. The currently described assay showed no effect of plant inhibitors when crude extracts of artificially infected taro, orchid plant materials, and naturally infected taro (prepared using Plant Lysis Kit)^25^ were tested using the developed LAMP assay (Figs. 8, 9, 10). In contrast to other developed diagnostic methods^37,38^, this *D. fangzhongdai* LAMP assay is rapid, requiring only about 15–30 min from sample preparation to detection and does not require sophisticated lab equipment (Fig. 7C).

High sensitivity is a prerequisite for a diagnostic assay that eliminates the probability of false negatives that could have serious negative consequences if a pathogen enters and becomes established in new geographical locations. Therefore, we evaluated the detection limit of the *D. fangzhongdai* LAMP assay in the presence of three hosts (taro, onion, orchid) matrices. The 5 µl of crude extract of either taro, onion, or orchid was added to preparations of tenfold serially diluted genomic *D. fangzhongdai* DNA, but no effect of host matrices was observed, and the detection limit remained at 100 fg, which is equivalent to 18–20 genome copies (Figs. 4A–C, 5). These results indicate that the developed assay is highly sensitive, robust, and specific. In comparison, endpoint PCR was performed with the primer set designed using the same unique MFS transporter gene region added to tenfold serially diluted DNA of *D. fangzhondai.* The target pathogen was detected in all dilutions down to 10 pg (Fig. 4D). An additional spiked assay was performed to assess inhibition by adding 1 µl of plant genomic DNA extracted from healthy taro corm to each PCR reaction mixture containing 1 µl of tenfold serially diluted DNA of *D. fangzhondai* (Fig. 4E). A 10 pg limit of detection was maintained, but it was 100-fold less sensitive than LAMP detection.

Evaluation of the LAMP reaction by multiple operators on the Rotor-Gene platform provided confidence in detection consistency, robustness, and sensitivity (Fig. 6). Assay performance levels were achieved on multiple operating devices, showing consistency across differing amplification platforms (Fig. 7). Finally, our assay was not inhibited by the presence of plant cellular matrices, indicating that assay functionality is not necessarily dependent on sample purity and suggesting that less-stringent extraction methods may be used. These findings suggest that the LAMP assay can be used with confidence in laboratories as well as at point-of-need.

## Methods

Any plant and plant materials used in this research comply with international, national and institutional guidelines.

### Target gene selection

Complete genomes of *Dickeya* species and *Pectobacterium* species were retrieved from the NCBI GenBank genome database and aligned using the progressiveMauve algorithm in Geneious Prime ver. 2021.2.2. Local collinear blocks (LCB) formed from homologous regions of three strains of *Dickeya fangzhongdai* (QZH3, PA1, and ND14B), one strain of *D. dadantii* subsp. *dieffenbachiae* (NCPPB 2976), and one strain of *Pectobacterium atrosepticum* (SCRI1043), were sifted for regions having potential for diagnostic development. Potential target regions exclusive to *D. fangzhongdai* were identified; regions were verified to be unique and universal to the species via NCBI GenBank nucleotide BLAST (https://blast.ncbi.nlm.nih.gov). Potential target genes having high average and median similarity among *D. fangzhongdai* genomes were considered for further endpoint PCR examination (data not shown). Primers for endpoint PCR were developed using Primer3 v4.1.0 using methods described by Arif and Ochoa-Corona^39^. Primer specificity was verified using the aforementioned whole genomes of *D. fangzhongdai* via Geneious Prime, and all available nucleotide sequences in the NCBI database through Primer-BLAST*.*

### LAMP primer design

The veracity of potential target regions were verified by BLAST and twelve publicly available (retrieved from the NCBI GenBank genome database) complete and draft genomes of *D. fangzhongdai* and one whole genome sequence representative of strains in Hawaii (Mohammad Arif, unpublished information; see Table 1)*. Dickeya* species and *Pectobacterium* species (Table 3) were used to validate the specificity of the designed primer sets for potential target genes using end-point PCR, and as a result, the major facilitator superfamily (MFS) transporter was selected for LAMP primer development.

Six LAMP primers, forward inner primer, forward outer primer, backward inner primer, backward outer primer, forward loop primer, and backward loop primer, were developed using PrimerExplorer V5 (https://primerexplorer.jp/e/) for the unique region of MFS transporter gene after the specificity of the endpoint primers were validated in vitro. Primer sequences were adjusted manually as necessary to avoid variable regions. All primers used are listed in Table 2.

### DNA extraction

Bacterial strains used for this study were streaked from glycerol stocks stored cultures at − 80 °C onto Dextrose Peptone Agar (DPA, peptone 10 g l^−1^, dextrose 5 g l^−1^, and agar 17 g l^−1^) or Nutrient Agar (NA) (BD, Becton Dickinson), and incubated at 26–28 °C for 1 or 2 days depending on the bacterial species. Bacterial gDNA was extracted from a pure culture according to the manufacturer’s instruction (DNeasy Blood and Tissue Kit, Qiagen, Germantown, MD). Plant gDNA was extracted from 100 mg of healthy tissues of orchid (*Phalaenopsis* sp.), onion (*Allium cepa*), and taro corm (*Colocasia esculenta*) using a DNeasy Plant Mini Kit (Qiagen).

### Endpoint PCR and LAMP assays

Each LAMP reaction was composed of 15 μl Isothermal Mastermix (Optigene, West Sussex, UK), 2 μl of LAMP primer mix (1.6 μM of Df-FIP and Df-BIP, 0.2 μM of Df-F3 and Df-B3, and 0.4 μM of Df-LF and Df-LB), and 1 μl of template DNA; reactions were brought to a final volume of 25 μl with nuclease free water. LAMP reactions and subsequent melt-curve analyses were conducted using a Rotor-Gene Q (Qiagen). In this study, the *D. fangzhongdai* type strain CFBP8607^T^ was used as the positive control and template-less reactions were added as negative controls. The amplification temperature of the reaction was set to 65 °C for 20 min. Melting curve analysis took place (99–80 °C) with increments of 0.2 °C. Amplification curves measuring fluorescence were analyzed by Rotor-Gene Q Software 2.3.1.49 to determine positive or negative calls. Melting curve data was used to further validate such results. Results were visually assessed after melting curve analysis by the addition of 3 μl of SYBR Green I (Molecular Probes Inc.) into each tube for result corroboration with fluorescent detections. Positive LAMP results appeared green under natural light and fluorescent during exposure to UV light after the addition of the dye. Negative results appeared orange under natural light, and fluorescence was absent during exposure to UV light.

Endpoint PCR amplification was used to validate the uniqueness of the primer pair designed to amplify a region of the MFS transporter gene. A subset of bacterial strains and plant samples from the LAMP inclusivity and exclusivity panels were selected for the target validation with endpoint PCR. Each 20 μl of endpoint PCR reaction consisted of 10 μl of 2× GoTaq Green Master Mix (Promega), 1 μl of both 5 μM AD-F1 and AD-R1 primers, 1 μl of template DNA, and 7 μl of nuclease-free water. The cycle conditions of the reaction included an initial denaturation of 95 °C for 3 min, followed by 35 cycles of denaturation at 95 °C for 20 s, annealing at 57 °C for 30 s, extension at 72 °C for 20 s, and a final extension at 72 °C for 3 min. PCR amplification of the MFS gene was conducted in a Bio-Rad T100 thermal cycler (Bio-Rad, Hercules, CA), and 15 μl of PCR amplicon was loaded into a 1.5% agarose gel stained with ethidium bromide (Invitrogen, Carlsbad, CA). Gel electrophoresis conditions were 100 V for 45 min. The PCR results were visualized under UV light using a gel documentation system (Bio-Rad Gel Doc-XR+).

### Specificity assays

The specificity of the developed LAMP primer set was demonstrated using the extensive and complete inclusivity and exclusivity panels (Table 3). The inclusivity panel consisted of the *D. fangzhongdai* type strain, CFBP8607^T^, isolated from pear in China and 6 *D. fangzhongdai* strains collected from taro in Hawaii. The exclusivity panel, which was composed of 94 non-target gDNA samples, consisted of phylogenetically close bacterial species in the genera *Dickeya* and *Pectobacterium*, plant associated niche-sharing bacterial species of additional genera, and gDNA isolated from typical *D. fangzhongdai* habitat matrices. Habitat matrices consisted of soil and expected host plant species including potato, orchid, and taro. LAMP reactions were prepared according to the specifications described above. The complete inclusivity panel and a subset of the exclusivity panel were also assayed in validation of an endpoint primer set described above.

### Sensitivity and spiked sensitivity assays

The limit of the developed LAMP assay’s analyte sensitivity was demonstrated and compared with those of endpoint PCR reactions performed in parallel. The endpoint PCR and LAMP reactions were conducted using the same protocols as previously stated. Sensitivity assays were performed on purified genomic DNA extracted from colonies of the *D. fangzhondai* type strain, CFBP8607, to determine the limit of LAMP detection. DNA was quantified using a Qubit 4 fluorometer (Thermo Fisher Scientific, Waltham, MA), serially diluted tenfold from 10 ng to 1 fg with nuclease-free water and assessed for both reaction types. The endpoint and LAMP reactions were performed by the addition of 1 μl of diluted DNA into each reaction tube.

Inhibitor resistance and the background impacts of plant tissues on the LAMP assay were demonstrated by performing sensitivity assays on gDNA spiked with plant crude lysates. Crude lysates were prepared from artificially infected orchid (stem), taro (corm), and onion (leaf) tissue; 1 g of tissue from each plant was processed using the Plant Material Lysis Kit (Optigene, Sussex, UK). Five μl of plant lysate was added to each tube with 1 μl serially diluted genomic DNA of *D. fangzhondai* CFBP8607^T^ (1 fg–10 ng) as used above.

The interference of plant genomic DNA with the accuracy of endpoint PCR analyte detection was examined. One μl of plant genomic DNA extracted from healthy taro corm or orchid leaf was added into each PCR reaction mixture containing 1 µl of tenfold serially diluted genomic DNA of *D. fangzhondai* (1 fg–10 ng). A negative control lacking bacterial DNA but containing extracted genomic taro or orchid DNA was used to confirm the primer specificity.

### Multi-operator blind tests

Multi-operator tests were performed by five independent teams at the University of Hawaii at Manoa to assess the reproducibility of the developed *D. fangzhongdai* LAMP assay. Each operator performed a blind test with eleven bacterial genomic DNA samples including 3 *D. fangzhongdai* strains, 7 other *Dickeya* species, and 1 *Pantoea* species from the inclusivity and exclusivity panels (Table 3), and a non-template negative control (water). The samples were arranged randomly without bias and undisclosed to the operators. LAMP assays were performed as previously described.

### Multi-device detection

The LAMP assay reactions were performed in triplicate to test the efficacy of three different heating devices (Qiagen Rotor-Gene Q, Bio-Rad 100 thermocycler, and dry bath). Eleven fresh bacterial colonies, including *D. fangzhongdai* strains (CFBP8607^T^ and PL145-PL150) and *D. aquatica* (LMG27354), *D. lacustris* (LMG30899), *D. undicola* (LMG30903), and *D. oryzae* (A5410) were grown on DPA/NA medium. Colonies were suspended in PCR tubes containing 100 μl of nuclease free water, boiled using the thermocycler at 95 °C for 10 min, and cooled; cooled colonies were centrifuged at full speed for 2 min. One μl of each supernatant was used as a template for LAMP reactions. The thermocycler conditions for LAMP assay were the same as the dry bath, 65 °C for 20 min.

### LAMP assay validation using artificially and naturally infected samples

Diagnostic capability and robustness of the developed LAMP assay were demonstrated using artificially infected taro and orchid samples, and naturally infected taro corms. The inoculum of four *D. fangzhongdai*, A6317 and PL145-147, 3 other species of *Dickeya*, LMG30903, LMG25990, A5410, and a *Pectobacterium* sp. LMG2404 were prepared from overnight culture and inoculated onto taro corm and orchid stem slices as follows.

Taro corms and orchid stems were cleaned using tap water and submerged in 0.6% hypochlorite for 3 min followed by three washes with sterile water. A loopful (~ 10 μl) of overnight grown bacterial culture was stabbed inoculated into each taro and orchid slice, and the slices were incubated in petri dishes at room temperature for 12–18 h. A total of 100 mg tissue from each inoculated slice was macerated and used for crude DNA isolation using a Plant Material Lysis Kit (Optigene). Five μl of crude DNA isolated from artificially infected samples were deployed for the LAMP assay. Genomic DNA of *D. fangzhongdai* and healthy taro corm/orchid stem lysates were treated as positive and negative controls, respectively.

## Supplementary Information

Supplementary Figure S1.

Supplementary Information 2.

## Data Availability

The genomes were retrieved from NCBI GenBank database and are available with following accession numbers: *D. fangzhongdai* QZH3 (NZ_CP031507) https://www.ncbi.nlm.nih.gov/nuccore/NZ_CP031507, *D. fangzhongdai* ND14b (NZ_CP009460) https://www.ncbi.nlm.nih.gov/nuccore/NZ_CP009460, *D. fangzhongdai* PA1 (NZ_CP020872) https://www.ncbi.nlm.nih.gov/nuccore/NZ_CP020872, *D. aquatica* 174/2^T^ (NZ_LT615367) https://www.ncbi.nlm.nih.gov/nuccore/NZ_LT615367, *D. chrysanthemi* Ech1591 (NC_012912) https://www.ncbi.nlm.nih.gov/nuccore/NC_012912, *D. dadantii* NCPPB 2976^T^ (NZ_CM001978) https://www.ncbi.nlm.nih.gov/nuccore/NZ_CM001978, *D. dianthicola* ME23 (NZ_CP031560) https://www.ncbi.nlm.nih.gov/nuccore/NZ_CP031560, *D. lacustris* S29^T^ (QNUT00000000) https://www.ncbi.nlm.nih.gov/nuccore/QNUT00000000, *D. oryzae* ZYY5^T^ (SULL00000000) https://www.ncbi.nlm.nih.gov/nuccore/SULL00000000, *D. parazeae* Ech586 (NC_013592) https://www.ncbi.nlm.nih.gov/nuccore/NC_013592, *D. poaceiphila* NCPPB 569^T^ (NZ_CP042220) https://www.ncbi.nlm.nih.gov/nuccore/NZ_CP042220, *D. solani* IPO 2222 (NZ_CP015137) https://www.ncbi.nlm.nih.gov/nuccore/NZ_CP015137, *D. undicola* 2B12 (JSYG00000000) https://www.ncbi.nlm.nih.gov/nuccore/JSYG00000000, *D. zeae* PL65 (NZ_CP040817) https://www.ncbi.nlm.nih.gov/nuccore/NZ_CP040817, *P. parmentieri* RNS 08-42-1A^T^ (NZ_CP015749) https://www.ncbi.nlm.nih.gov/nuccore/NZ_CP015749, *P. brasiliense* 1692 (NZ_CP047495) https://www.ncbi.nlm.nih.gov/nuccore/NZ_CP047495, and *P. carotovorum* XP-13 (NZ_CP063242) https://www.ncbi.nlm.nih.gov/nuccore/NZ_CP063242.
