# Supplementary material for: Loop-mediated isothermal amplification (LAMP) assay for specific and rapid detection of Dickeya fangzhongdai targeting a unique genomic region
Source: Sci Rep. 2022 Nov 10;12:19193. doi: 10.1038/s41598-022-22023-4 (PMC9649655; doi:10.1038/s41598-022-22023-4)
Supplement: Supplementary file 1 — Supplementary Figure S1. [file 41598_2022_22023_MOESM1_ESM.pdf]

Loop-mediated isothermal amplification (LAMP) assay for specific and rapid detection of *Dickeya fangzhongdai* targeting a unique genomic region

Anuhea DeLude[1#], Riley Wells[2#], Sherine Boomla[2#], Shu-Cheng Chuang[1#], Frank Urena[2,7#], Aaron Shipman[1#], Noelle Rubas[2#], Donna Lee Kuehu[2,7#], Buster Bickerton[2#], Taylor Peterson[3#], Shefali Dobhal[1], Dario Arizala[1], Diksha Klair[1], Francisco Ochoa-Corona[4], Md Emran Ali[5], Jenee Odani[3], Jon-Paul Bingham[2], Daniel Jenkins[2], Jacqueline Fletcher[4], James P. Stack[6], Anne M. Alvarez[1] & Mohammad Arif[1]*

[1]Department of Plant and Environmental Protection Sciences, University of Hawaii at Manoa, Honolulu, HI, USA.
[2]Department of Molecular Biosciences and Bioengineering, University of Hawaii at Manoa, Honolulu, HI, USA.
[3]Department of Human Nutrition, Food and Animal Sciences, University of Hawaii at Manoa, Honolulu, HI, USA.
[4]Institute for Biosecurity & Microbial Forensics, Oklahoma State University, Stillwater, OK, USA.
[5]Department of Plant Pathology, University of Georgia, Tifton, GA, USA.
[6]Department of Plant Pathology, Kansas State University, Manhattan, KS, USA.
[7]Department of Cell and Molecular Biology University of Hawaii at Manoa, Honolulu, HI, USA.

[#]These authors contributed equally to this work

*Corresponding author: arif@hawaii.edu; Phone +1 808-956-7765

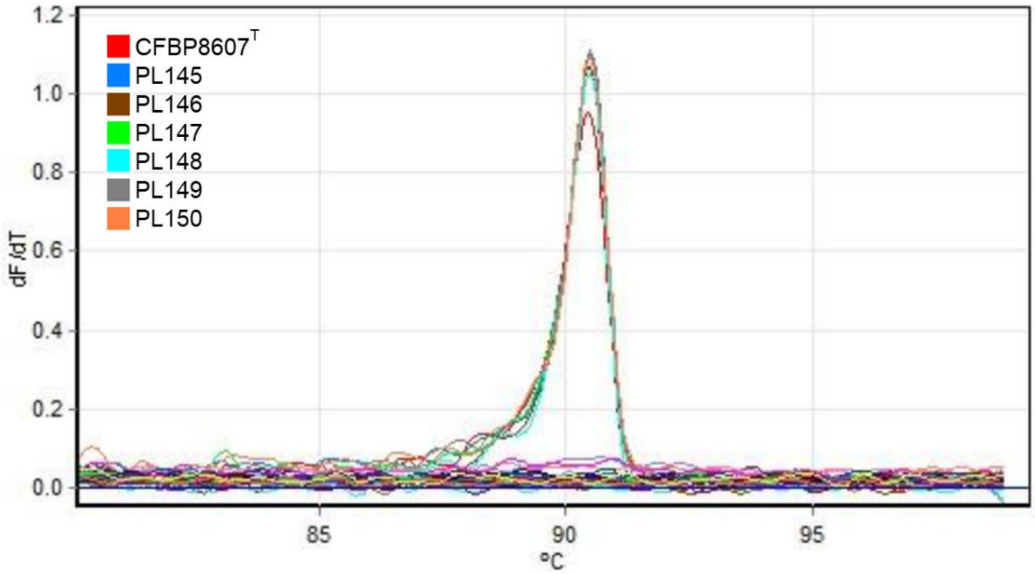

Figure S1. Melting curve results of Loop Mediated Isothermal Amplification (LAMP) assay designed for *D. fangzhongdai* detection. Thirty-five bacterial strains and water as a non-template control were tested. The colored peaks (>0.2 dF/dT) indicated the seven *D. fangzhongdai* strains, CFBP8607^T, PL145, PL146, PL147, PL148, PL149, and PL150 presenting the positive results by showing the peaks. All non-*D. fangzhongdai* showed no peaks crossing 0.2dF/dT.
