# Supplementary material for: Loop-mediated isothermal amplification (LAMP) assay for specific and rapid detection of Dickeya fangzhongdai targeting a unique genomic region
Source: Sci Rep. 2022 Nov 10;12:19193. doi: 10.1038/s41598-022-22023-4 (PMC9649655; doi:10.1038/s41598-022-22023-4)
Supplement: Supplementary file 2 — Supplementary Information 2. [file 41598_2022_22023_MOESM2_ESM.pdf]

Original Full Gel Picture

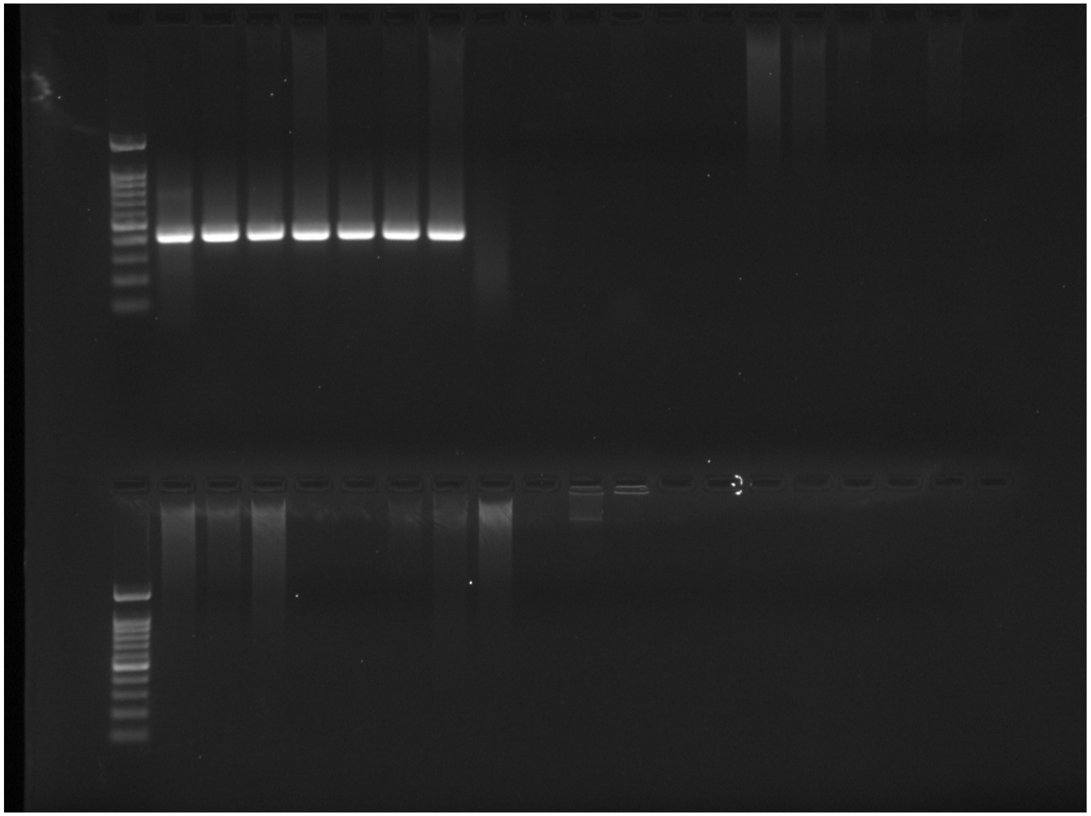

Figure 3. The specificity validation of the endpoint PCR primer pair designed to specifically detect *Dickeya fangzhongdai*.

Original Full Gel Picture

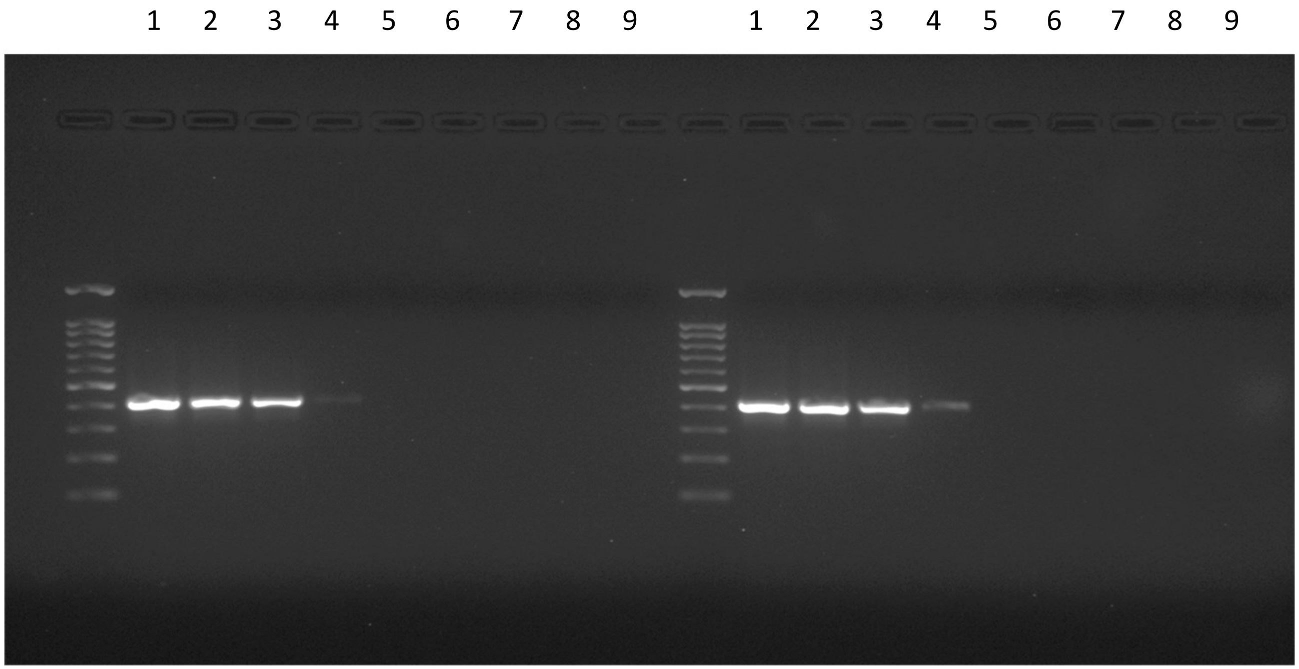

Figure 4. Detection limit determination of loop mediated isothermal amplification (LAMP) assay designed for specific detection of *Dickeya fangzhongdai.*
(D) Endpoint sensitivity assay with genomic DNA, serially diluted 10-fold (from 10 ng to 1 fg);
(E) spiked assay (1 µl of healthy taro corm genomic DNA was added in 10-fold serially diluted genomic DNA).
